# Fine-tuning of mononuclear phagocytes for improved inflammatory responses: role of soybean-derived immunomodulatory compounds

**DOI:** 10.3389/fnut.2024.1399687

**Published:** 2024-05-24

**Authors:** Hiroyuki Tezuka, Shinjiro Imai

**Affiliations:** ^1^Department of Cellular Function Analysis, Research Promotion Headquarters, Fujita Health University (FHU), Aichi, Japan; ^2^School of Bioscience and Biotechnology, Tokyo University of Technology, Tokyo, Japan; ^3^Institute of Metabolic Function, Kanagawa, Japan

**Keywords:** allergy, anti-inflammation, dendritic cells, macrophages, mononuclear phagocytes, phytochemicals, soybeans

## Abstract

The concept of inflammation encompasses beneficial and detrimental aspects, which are referred to as infectious and sterile inflammations, respectively. Infectious inflammation plays a crucial role in host defense, whereas sterile inflammation encompasses allergic, autoimmune, and lifestyle-related diseases, leading to detrimental effects. Dendritic cells and macrophages, both of which are representative mononuclear phagocytes (MNPs), are essential for initiating immune responses, suggesting that the regulation of MNPs limits excessive inflammation. In this context, dietary components with immunomodulatory properties have been identified. Among them, soybean-derived compounds, including isoflavones, saponins, flavonoids, and bioactive peptides, act directly on MNPs to fine-tune immune responses. Notably, some soybean-derived compounds have demonstrated the ability to alleviate the symptom of allergy and autoimmunity in mouse models. In this review, we introduce and summarize the roles of soybean-derived compounds on MNP-mediated inflammatory responses. Understanding the mechanism by which soybean-derived molecules regulate MNPs could provide valuable insights for designing safe immunomodulators.

## 1 Introduction

Inflammation serves as an immune response to infection or sterile tissue damage. Infectious inflammation is largely mediated by innate immunity against infectious agents, such as pathogen-associated molecular patterns, which contributes to the early host defense (1). In this process, mononuclear phagocyte (MNP)-derived reactive oxygen species (ROS) are essential for eliminating invading pathogens, often accompanied by collateral tissue damages. Paradoxically, upon the elimination of pathogens, inflammation promotes wound healing and tissue remodeling, leading to beneficial effects on the host (1). Conversely, sterile inflammation is mediated by both innate and adaptive immunity against self-antigens, such as altered organ-specific antigens and damage-associated molecular patterns (also known as alarmin) released by injured or dying cells, as well as harmless exogenous antigens, including dietary proteins and airborne pollens. This type of inflammation causes allergic, autoimmune, and lifestyle-related (non-communicable) diseases, which lead to long-term detrimental effects (1, 2). Therefore, the sterile chronic inflammatory diseases are thought to be impaired immune tolerance or suppression against self-antigens and harmless exogenous antigens, which are tightly regulated by MNPs.

Dendritic cells (DCs) and macrophages, both of which are representative MNPs, share the capacity of antigen-presentation to T cells. These MNPs are distributed in almost all organs and tissues, playing an essential role in inducing immunity to non-self-antigens and promoting tolerance to self-antigens as well as harmless exogenous antigens (3, 4). DCs are the specialized phenotype, stimulating antigen-specific naïve T cells and functioning in both innate and adaptive immunity. In contrast, macrophages activate DC-induced antigen-experienced effector T cells and participate in innate rather than adaptive immunity (3, 4). Therefore, the functional impairment of MNPs leads to inappropriate immune responses against antigens, contributing to the onset of various inflammatory diseases (3, 4).

Sterile inflammations are associated with genetic predispositions, which can be triggered by physical, chemical, and metabolic stimuli. Interestingly, the prevalence of sterile inflammations, especially autoimmune diseases, is much higher in Western countries than in Asian countries, highlighting the significance of the Western lifestyle as an environmental risk factor (5). Habitual intake of high-calorie Western diets, which contain low amounts of plant-derived components, such as fibers and antioxidants, is thought to exacerbate inflammatory diseases (6). In this context, although soybeans (*Glycine max*) are an important nutrient as a major source of plant-based protein, which are being consumed worldwide (7), fermented soybean products, including soy source, natto, miso, tofuyo, douchi, tempe, thua nao, and sufu, are preferentially consumed in Asian countries (8). Consistent with these reports, a large cohort study revealed that the habitual intake of soybeans and/or fermented soybean products can prevent the development of cardiovascular diseases, including atherosclerosis (9). Furthermore, accumulating evidence suggests that soybeans contain various immunomodularoty compounds with pro- and anti-inflammatory properties, including isoflavones, saponins, and bioactive peptides. These compounds act directly on MNPs to finely regulate immune responses in a context-dependent manner (10). Furthermore, dietary soybean-derived compounds have been demonstrated to prevent inflammatory responses in allergen- or self-antigen-sensitized mice (11–14), implying that these compounds regulate the function of MNPs that reside in the intestine, the primary site for digestion and absorption of dietary components. Notably, the intake of soybeans and some soybean-derived compounds has been shown to improve the composition and diversity of gut microbiota and increase the relative abundance of short-chain fatty acid-producing bacteria that are involved in inducing anti-inflammatory immune responses in mammals, indicating that soybean-derived compounds indirectly impact immune cells by altering the microbiota (15–17).

Considering the pivotal role of MNPs in the onset of various inflammatory diseases and the regulatory effect of soybean-derived compounds on MNPs, these compounds may serve as novel immunomodulators targeting MNPs. Here, we present a comprehensive review and discussion of the current understanding of the immunomodulation via MNPs by soybean-derived compounds, largely based on the knowledge obtained from studies on mouse models.

## 2 Mononuclear phagocytes

MNPs comprise DCs, macrophages, and monocytes. Among them, DCs and macrophages share common functionalities in host defense, such as antigen recognition, uptake, processing, and presentation. However, MNPs exhibit differences in their origin, differentiation, phenotype, and localization, comprising heterogeneous populations.

### 2.1 Dendritic cells

DCs consist of two subsets: FMS-like tyrosine kinase ligand-dependent DCs and colony-stimulating factor (CSF) 1/2-dependent monocyte-derived DCs. The former includes CD11c^hi^MHC class II^+^ conventional DCs (cDCs) and CD11c^int^MHC class II^lo^ plasmacytoid DCs (pDCs) (3). Furthermore, cDCs can be divided into type 1 (cDC1) and type 2 cDCs (cDC2) based on the expression of cell surface markers and transcription factors, as described elsewhere (4).

Upon engulfing antigens, including bacteria, cDCs migrate into the regional lymph nodes through afferent lymphatic ducts in a chemokine (C-C motif) receptor 7 (CCR7)-dependent manner. After arriving at the lymph nodes, cDCs prime antigen-specific naive T cells via antigen presentation and costimulation (3, 4). During this process, “immunogenic” cDCs secrete various cytokines that induce the differentiation of naïve CD4^+^ T cells into mature helper T (Th) cells. For example, interleukin (IL)-12, IL-4, and IL-23 selectively induce the differentiation into Th1, Th2, and Th17, respectively. Furthermore, cDC-induced antigen-specific Th cells promote the differentiation of B cells into antibody-producing plasma cells, facilitating antigen eliminations. In contrast, “tolerogenic” regulatory cDCs conditioned under an anti-inflammatory environment secrete transforming growth factor (TGF)-β1 and IL-10 to induce regulatory T (Treg) cells. Unlike cDCs, pDCs sensed antigens, including viruses, and then migrate into the lymph nodes through the bloodstream in a CCR9-dependent manner. Despite their lower antigen-presenting capacity compared to that of cDCs, pDCs can secrete substantial amounts of type-I interferons (IFNs) to eliminate viruses and virus-infected cells (3, 4).

Monocyte-derived DCs spread into inflamed tissues in a CCR2-dependent manner. Upon infection with *Listeria monocytogenes*, tumors necrosis factor (TNF)-α and inducible nitric oxide (NO) synthase-producing DCs (Tip-DCs), a subset of monocyte-derived DCs, are predominantly induced at the site of infection in a CCR2- and myeloid differentiation primary response 88 (MyD88)-dependent manner, leading to the initiation of Th1-type immune responses to eliminate the bacteria (18, 19). Interestingly, we found that, under steady-state conditions, “naturally occurring” Tip-DCs that promote immunoglobulin A production are induced in the small intestine by toll-like receptor (TLR)-dependent recognition of microbiota (20). This subset also originates from monocytes, which are recruited into the intestine in a CCR2- and MyD88-dependent manner (20, 21).

### 2.2 Macrophages

Macrophages are divided into tissue-resident and monocyte-derived subsets based on their ontogeny, localization, and functions (22). Tissue-resident macrophages, which originate from progenitors in the embryonic yolk sac and fetal liver, populate various organs, including the brain, dermis, liver, kidney, lung, and intestine, before birth. These tissue-resident subsets perpetuate their numbers through self-renewal to maintain tissue homeostasis (22). On the other hand, monocyte-derived subsets originate from common monocyte progenitors in the bone marrow after birth (23). Notably, under stady-state conditions, some tissue-resident macrophages in the organs, excluding the brain, are replaced by monocyte-derived macrophages during aging in a CSF1-dependent manner (22). In inflammatory contexts, monocytes selectively migrate into inflamed tissues in a CCR1/CCR2/CCR5/CX3CR1-dependent manner, where they generally differentiate into M1 macrophages, as described below.

Numerous culture experiments have demonstrated that F4/80^+^CD11b^+^ activated macrophages are divided into “classically” M1 and “alternatively” M2 subsets based on their functional and phenotypic properties (24). M1 macrophages induced by stimulation with IFN-γ and lipopolysaccharides (LPS), a cell wall component from Gram-negative bacteria, represent a pro-inflammatory “immunogenic” phenotype. This subset initiates immune responses against invading pathogens or tumors by producing pro-inflammatory factors, such as TNF-α, NO, and ROS, in a nuclear factor kappa B (NF-κB) signaling-dependent manner (24, 25). In contrast, M2 macrophages induced by Th2 cell-derived cytokines, such as IL-4 and IL-10, represent an anti-inflammatory “tolerogenic” phenotype. This subset promotes the remodeling of damaged tissues by producing anti-inflammatory mediators, such as TGF-β1 and IL-10, thereby resolving inflammation (24). Simultaneously, M2 subsets promote the remodeling of damaged tissues by producing ornithine, a substrate of polyamine synthesis. Nowadays, anti-inflammatory M2 macrophages can be categorized into at least four subsets (M2a, M2b, M2c, and M2d) based on the expression of transcription factors (26). Given that M2 macrophages play a role in repairing M1 macrophage-mediated tissue damages, their appearance in damaged tissues is likely attributable to be either the reprogramming from M1 to M2 subsets or the replenishment of M2 subsets during the recovery process. Although the concept of reprogramming seems plausible, the mechanisms underlying the reprogramming toward M2 subsets remain unclear.

## 3 Role of mononuclear phagocytes in inflammatory diseases

MNPs recognize various antigens through pattern-recognition receptor TLRs and scavenger receptor C-type lectins. These receptors activate ubiquitous transcription factor NF-κB via various intracellular signaling pathways, including MyD88, mitogen-activated protein kinase (MAPK), and phosphoinositide 3-kinase (PI3K)/Akt (also known as protein kinase B) (25). Once activated, NF-κB is recruited into the nucleus, binding to the promoter regions of genes encoding inflammatory factors, including pro-inflammatory cytokines and the NOD-like receptor family, pyrin domain-containing 3 inflammasome complex, leading to the induction of immunogenic MNPs (25). Therefore, the regulation of ubiquitous transcription factor in MNPs could prevent aberrant inflammation. In this section, we describe the significance of MNPs in inflammatory diseases, drawing insights obtained from studies on mice with MNP-specific disruption of the NF-κB family, which is the master regulator of immune responses.

Macrophage-specific deficiency of inhibitory κB kinase β (IKKβ), an NF-κB activating kinase, protected mice from myelin oligodendrocyte glycoprotein-induced experimental autoimmune encephalomyelitis (EAE), a model of multiple sclerosis, due to a reduced number of Th1 and Th17 cells, along with an increased number of Treg cells in the spinal cord (27). Macrophage-specific deletion of IKKβ in low-density lipoprotein receptor-deficient mice led to reduced high-fat diet-induced atherosclerosis (28). These findings indicate that, upon recognition of self-antigens, prolonged NF-κB activation in MNPs induces inflammatory diseases through the activation of pathogenic T cell subsets. Conversely, macrophages-specific IKKβ- or p65-deficient mice showed increased sensitivity to LPS-induced shock due to elevated production of pro-inflammatory cytokines, including IL-1β, IL-6, and TNF-α (29, 30). These findings suggest that NF-κB signaling in MNPs limits the aberrant inflammation against infectious agents. Therefore, the regulation of MNPs with dual roles in inducing immunity to non-self-antigens and tolerance to self-antigens is dependent on NF-κB signaling. This signaling pathway serves as a target for soybean-derived compounds, as described below.

## 4 Soybean-derived compounds that regulate MNP function

Soybeans have long been recognized for their nutritional value and health benefits (7–9). In addition to their well-established effects on cardiovascular health (31) and cholesterol levels (32), soybean-derived compounds influence on the immune system (10, 33). Among these compounds, isoflavones, saponins, flavonoids, peptides, proteins, lipids, vitamins, and minerals have been confirmed to have immunomodulatory effects. This section focuses on the interplay between the various soy compounds and MNPs (Table 1), highlighting the potential immunomodulatory effects that may contribute to overall health.

**Table 1:** Soybean-derived compounds that regulate mononuclear phagocytes.

	Compounds	MNPs (Stimuli)	MNP’s function		Target molecules	*In vivo* models (improved)	Ref.
Isoflavones	Genistein	MoDCs (CT)	IL-6, IL-8, IL-9, IL-13, CD80, CD83	↓	-	Allergy	(11)
	Genistein	MoDCs (LPS)	IL-1β, TNF-α, IL-12, MHC I, CD83, CD86	↓	-	-	(34)
	Isoflavones glycosides	RAW (LPS)	IL-1β, IL-6, TNF-α, MCP-1, PGE2, NO, ROS	↓	NF-κB	Colitis	(35)
	Protodaidzeone	THP-1 (LPS)	IL-6, IL-12, TNF-α	↓	-	Allergy	(36)
Saponins	Sayasaponin Ab	Pec Mφ (LPS)	IL-6, TNF-α, PGE2, NO	↓	TLR4/NF-κB	Colitis	(37)
	Soyasaponin	RAW (LPS)	PGE2, ROS	↓	PI3K/Akt/NF-κB	-	(38)
	Sayasaponin I	RAW (LPS)	IL-1β, TNF-α, NO, PGE2	↓	NF-κB	Colitis	(39)
Flavonoids	Apigenin	RAW (LPS)	TNF-α, NO	↓	-	-	(40)
	Anthocyanin (Extracts)	RAW (LPS)	IL-6, TNF-α, MCP-1, PGE2, NO, ROS	↓	MAPK	-	(41, 42)
Peptides	Crude	RAW (LPS)	IL-1β, IL-6, TNF-α, TLR4	↓	MAPK/NF-κB	-	(43)
	Lunasin	RAW (LPS)	IL-1β, IL-6, NO, PGE2	↓	NF-κB	-	(44)
	Lunasin	RAW (−)	IL-6, Phagocytosis	↑	-	-	(45)
	Lunasin (enriched)	RAW (−)	IL-1β, IL-6, NO, ROS, Phagocytosis	↑	-	-	(46)
	Lunasin	MoDCs (−)	IL-1β, IL-6, TNF-α, CCL3, CCL4, NO, ROS, CD40, CD86	↑	-	-	(47)
Lipids	PI	THP-1 (LPS)	IL-1β, MCP-1	↓	NF-κB	-	(48)
	PC	Pec Mφ (−)	Phagocytosis	↑	-	-	(49)
Poly-saccharides	(Soy hull)	RAW (−)	IL-1β, IL-6, TNF-α, IL-12, NO, ROS, CD40, Pinocytosis	↑	TLR2/ MAPK/NF-κB	-	(50)
	(Soy sauce)	RAW (−)	IL-6, TNF-α, IL-12, NO	↑	MAPK/NF-κB	-	(51)

CT, cholera toxin; LPS, lipopolysaccharide; MNPs, mononuclear phagocytes; MoDCs, monocyte-derived dendritic cells; NO, nitric oxide; PC, phosphatidylcholine; Pec Mf, peritoneal macrophages; PI phosphatidylinositol; RAW, RAW264.7 cell lines; ROS, reactive oxygen species.

### 4.1 Isoflavones

**Figure 1:**
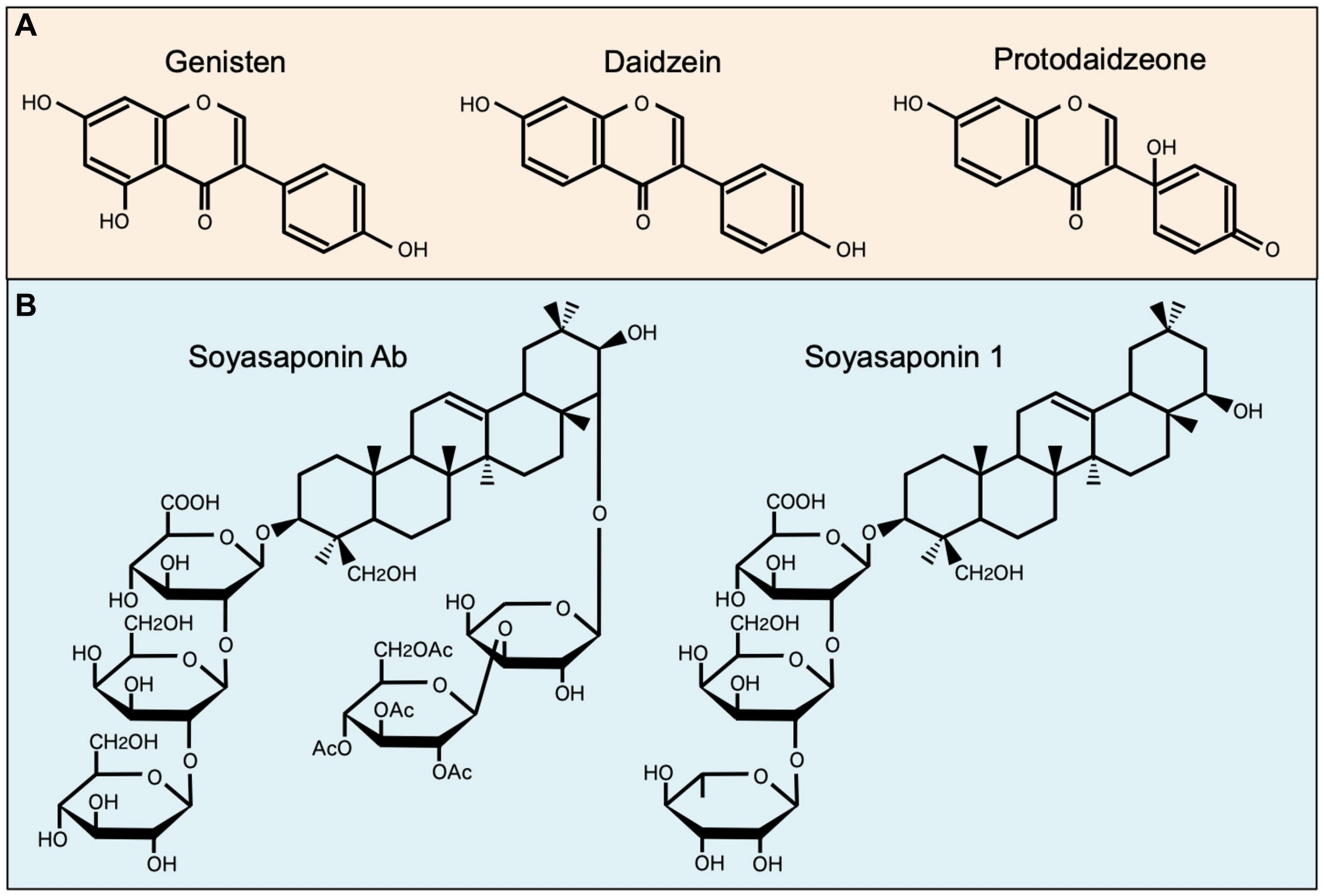
Chemical structures of representative soybean-derived compounds with immunomodulatory properties. The structures of isoflavone aglycones **(A)** and soyasaponins **(B)** is shown for comparison.

Among well-studied soybean-derived compounds, isoflavones standout bioactive compounds. Genistein and daidzein, the representative soy isoflavone aglycones, are synthesized from their glycosides genistin and daidzin, respectively, by gut microbial b-galactosidase (52). Notably, these isoflavone aglycones function as phytoestrogens with structural similarities to mammalian estrogens, acting as estrogen receptor modulators (53, 54) (Figure 1). However, recent studies have expanded their role beyond estrogen modulation, revealing their impact on MNPs, such as DCs and macrophages (11, 34, 35, 55, 56) (Table 1). Upon stimulation with LPS, these compounds regulate the production of inflammatory cytokines (IL-1β, IL-6, IL-12, and TNF-α), mediators (NO, prostaglandin E2 [PGE2], and ROS), phagocytosis, and differentiation in human monocyte-derived DCs and the murine macrophage cell line RAW264.7 cells, suggesting that isoflavones prevent the generation of immunogenic MNP subsets (11, 34, 35, 55, 56). Notably, dietary genistein skewed murine macrophage polarization toward M2-like subsets and improved clinical symptoms in a dextran sulfate sodium-induced colitis model (35, 56). Mechanistically, the immunomodulatory effect of isoflavone glycosides in water-soluble soybean extracts on murine MNPs is thought to be mediated by inhibiting NF-κB-signaling pathways through suppression of IKKα/β phosphorylation (35) (Table 1). Understanding the intricate interaction between soy isoflavones and MNPs is pivotal for elucidating the immunological mechanisms underlying soy isoflavone-mediated health effects and their potential applications in therapeutic interventions for various inflammatory diseases.

### 4.2 Saponins

Soy saponin is general term for oleanane triterpene (aglycone basic skelton) glycosides, of which there are more than 50 different molecular species. These diverse species originate from differences in aglycone structure, sugar chain, and sugar acetylation (57) (Figure 1). Soy saponins are naturally occurring compounds with diverse biological activities (57, 58). Some studies showed that soy saponins regulate the production of pro-inflammatory cytokines and mediators by murine macrophages (37, 38) (Table 1). For example, soy saponins inhibit the production of inflammatory mediators, such as IL-1β, TNF-α, NO, PGE2, and ROS, in LPS-stimulated peritoneal macrophages and RAW264.7 cells (37, 38), possibly prompting to polarization toward M2-like subsets. The soy saponin-induced polarization toward M2-like subsets is dependent on the suppression of TLR4-mediated activation of NF-κB, MAPK, and PI3K/Akt (37–39) (Table 1). Therefore, dietary saponins may contribute to the regulation of excessive immune responses.

Soy saponins exhibit structural diversity, as they are a diverse group of amphiphilic glycosides with distinctive chemical structures (Figure 1). Therefore, their immunomodulatory effects may vary depending on differences in chemical compositions. This is evident in the distinct actions of soyasoponin Ab compared to that of soyasaponin I in regulating macrophage function. Specifically, soyasaponin Ab inhibits the binding of LPS to the cell surface receptor TLR4 (37), whereas soyasaponin I directly inhibit the phosphorylation of IκBα (39) (Table 1). Understanding the structure–activity relationships of soy saponins is crucial for elucidating the molecular mechanism through which they influence the regulation of immune responses.

### 4.3 Flavonoids

Soy flavonoids, a diverse group of phytochemicals abundantly present in soy-based products, have garnered significant attention owing to their potential health benefits (59). Soy flavones, including apigenin and chrysin, prevent LPS-induced production of NO and TNF-α in RAW264.7 cells (40). In contrast, black soybean-derived anthocyanines prevent the production of pro-inflammatory cytokines (IL-6 and TNF-α), chemokines (MCP-1), and mediators (NO, PGE2, and ROS) by inhibiting MAPK/JNK/ERK/p38, upstream molecules of NF-κB, in LPS-stimulated murine macrophages (41, 42) (Table 1). In an adipocyte-macrophage coculture experiment, anthocyanins prevented murine macrophage-stimulated release of free-fatty acids and improved insulin resistance in adipocytes (42). These findings suggest that isoflavone-mediated polarization toward M2-like subsets improves sterile inflammatory diseases, including type 2 diabetes.

### 4.4 Bioactive peptides

Soy proteins, abundant in essential amino acids and bioactive peptides, play important roles in immune regulation (10). In an experiment using RAW264.7 cells, soy protein-derived crude low-molecular weight peptides inhibited LPS-induced production of M1-type cytokines (IL-1β, IL-6, and TNF-α) by suppressing MAPK/JUN pathways and IκB phosphorylation in NF-κB pathways (43). Recent findings revealed that hydrolyzed soy protein containing bioactive lunasin, a 43-amino acid peptide, inhibited LPS-induced secretion of M1-type mediators (IL-1β, IL-6, and NO) through suppressing NF-κB nuclear translocation in RAW264.7 cells (44). Contrary, lunasin enhances phagocytosis and induces the production of M1-type mediators in unstimulated RAW264.7 cells, contributing to the activation of immune responses (45, 46) (Table 1). Lunasin also exerts diverse effects on human monocyte-derived DCs, influencing their maturation, M1-type cytokine production, and antigen presentation, which potentiate adaptive immune responses as an adjuvant (47). These findings suggest that the regulation of macrophages by lunasin in the presence of LPS leads to opposing immune consequences.

### 4.5 Others

Soybean-derived lipids, including phospholipids and sterols, have been investigated for their immunomodulatory effects (60). A human study revealed that individuals on a soy oil-supplemented diet showed a tendency toward lower LPS-induced production of IL-6 and TNF-α in peripheral blood mononuclear cells than those on a control diet (61). This finding suggests the presence of unique fatty acids with immunomodulatory effects. Soy lipids contain a diverse array of compounds, including phospholipids, glycolipids, and triglycerides (60). Among them, soy phospholipids, particularly phosphatidylinositol, inhibit LPS-induced expression of IL-1β and MCP-1 through the suppression of IKK phosphorylation in the human monocytic cell line THP-1 (48). Additionally, soy lecithin (also known as phosphatidylcholine) influences murine macrophage function, affecting their phagocytic and antigen-processing abilities (49) (Table 1). Collectively, soy lipids presumably regulate NF-κB pathways in MNPs, influencing the expression of genes involved in inflammatory responses. The interplay between soy lipids and MNPs presents a fascinating avenue for research in the field of immunonutrition.

Polysaccharides derived from legumes exhibit various immunomodulatory functions (62). Particularly, soy polysaccharides induce the production of pro-inflammatory cytokines (IL-1β, IL-6, IL-12, and TNF-α) and mediators (NO and ROS) in unstimulated RAW264.7 cells (50, 51). Notably, the immunostimulatory effects are dependent on JUN/ERK/p38-mediated activation of NF-kB signaling (51) (Table 1). These findings suggest that soybean-derived immunostimulatory agents targeting MNPs offer protective effects against infections. Additionally, soybeans represent an abundant source of vitamins (vitamin E, D, and folate) and minerals (zinc), essential for maintaining immune responses (63–65). These micronutrients may directly or indirectly influence MNP functions by supporting cellular processes critical for antigen presentation and immune response regulation.

## 5 Soybean-derived compounds as therapeutic agents to control allergic inflammation

The higher prevalence of inflammatory diseases in Western countries than in Asian countries correlates well with the observed habitual intake of soybeans and their products (8). Soybean-derived compounds, including genistein, daidzein, soyasaponin, anthocyanins, phospholipids, and peptides, prevent LPS-induced production of inflammatory mediators in MNPs (34, 37, 38, 41–43, 48, 55), possibly achieved through the suppression of NF-κB pathways as described above. In this context, dietary isoflavones, including genistein and daidzein, reduce the anaphylactic symptoms in peanut antigen-sensitized mice, due to reduced DC maturation (11).

Our research revealed that a hot water extract of green soybeans significantly reduces the level of antigen-specific IgE in an ovalbumin-sensitized allergic mouse model and suppresses nasal secretion in a 2, 4-toluene diisocyanate-induced guinea pig rhinitis model (12). The human trial study consisted of two open-label treatment phases. In Part 1, participants were administered a daily single dose of green soybean extracts (3 g) over a 6-week period. In a trial study involving 16 patients with cedar pollinosis (8 men and 8 women aged 30 to 60 years) living in the Kanto region of Japan, those who continued to consume green soybeans after pollen dispersal experienced less severe symptoms of cedar pollinosis than those who had ceased consumption before pollen dispersal (12). This result suggests that green soybean extract holds considerable potential as an orally effective immunomodulator for treating various allergic diseases. Soybeans contain several bioactive compounds, such as genistein, daidzein, and saponins, which have been independently reported to possess anti-inflammatory and immunomodulatory properties. These compounds may partially explain the bioactivities of green soybean extracts. However, green soybeans have slightly higher levels of these compounds than in yellow soybeans (36). Additionally, there may be other unknown compounds present in green soybean extracts that contribute to their full range of bioactivities. Furthermore, we screened visible light-irradiated soybean extracts (LIEGS) inhibiting the production of cytokines. LIEGS strongly suppressed IL-2 production in Jurkat cells, with the effect being specific to green soybeans but not to other colored soybeans (36). LIEGS also suppressed LPS-induced expression of IL-6, IL-12, and TNF-α in THP-1 cells in a dose-dependent manner. Furthermore, dietary LIEGS reduced dermatitis scores and improved skin damages in NC/Nga mice, an atopic dermatitis model, possibly due to the suppression of pro-inflammatory cytokine production by MNPs (36). Finally, we successfully identified protodaidzeone, an isoflavone from LIEGS, as a novel dietary immunomodulator (36).

## 6 Perspectives

The multifaceted components of soybeans exert a complex yet intriguing influence on MNPs. The interactions between MNPs and isoflavones, proteins, saponins, lipids, and micronutrients contribute to the immunomodulatory potential of soybeans. A comprehensive understanding of these mechanisms may offer insights for developing dietary strategies to finely regulate immune responses and promote overall health. Immunomodulatory research centered on soybean-derived compounds holds significant potential for enhancing human health and combating various inflammatory diseases. Therefore, continued exploration of the mechanisms underlying the immunomodulatory effects of soybean-derived compounds and their therapeutic applications is crucial for harnessing their full potential. Although substantial preclinical research supports the immunomodulatory potential of soybean-derived compounds, further clinical trials and human studies are essential to validate these findings and assess their efficacy, safety, and optimal dosing regimens in diverse populations.

## Author contributions

HT: Conceptualization, Funding acquisition, Project administration, Supervision, Validation, Writing – original draft, Writing – review & editing. SI: Conceptualization, Validation, Writing – original draft, Writing – review & editing.
